# Triple Threat: A Case of Erysipelothrix rhusiopathiae Septicemia Complicated by Multi-Valvular Endocarditis, Spinal Osteomyelitis, and Septic Embolic Stroke

**DOI:** 10.7759/cureus.27789

**Published:** 2022-08-08

**Authors:** Emilio De Narvaez, David Schoenfeld, Amro Elshereye, Joann D Tran, Richard L Oehler

**Affiliations:** 1 Department of Internal Medicine, University of South Florida Morsani College of Medicine, Tampa, USA; 2 Division of Infectious Disease and International Medicine, University of South Florida Morsani College of Medicine, Tampa, USA

**Keywords:** gram-positive rods, stroke, septic embolic stroke, osteomyelitis, valvular endocarditis, endocarditis, erysipelothrix rhusiopathiae

## Abstract

*Erysipelothrix rhusiopathiae* is a Gram-positive rod associated with zoonotic infections, most commonly the soft tissues. It can be present in several ways, though most commonly as erysipeloid. Rarely, it may manifest systemically with septic organ involvement such as endocarditis or osteomyelitis. Here, we present the case of a 71-year-old male who presented to the hospital with back pain and neurological deficits. He was found to be bacteremic with *E. rhusiopathiae*, and imaging demonstrated the presence of multi-valvular endocarditis, spinal osteomyelitis with epidural abscess, and septic embolic stroke. Though such complications of *E. rhusiopathiae* septicemia have been documented in the literature, this is the first reported case of all three manifestations in one patient.

## Introduction

*Erysipelothrix rhusiopathiae* is a pleomorphic, encapsulated, Gram-positive, non-sporulating bacillus associated with zoonotic infections, most commonly of the soft tissues. Though most commonly associated with swine exposure, this bacterium is known to be present in a range of animals, both domestic and commercial [1]. Disease in humans has been described to follow three different clinical presentations. The most common is the localized cutaneous form, termed erysipeloid, which presents as cellulitis at the site of exposure. The diffuse cutaneous form involves spread to remote sites of the body with systemic symptoms and lesions ranging from those seen in erysipeloid to a more bullous pattern. Lastly, and most severely, is the systemic form characterized by septicemia [2]. Though cases of endocarditis, osteomyelitis, and even stroke from septic embolization have been described in the literature, there has not yet been a case reported that demonstrates all three manifestations [3-12]. Herein, we describe the case of a 71-year-old man who presented with thumb weakness and back pain. He was subsequently found to have a subacute ischemic cerebral infarct as well as acute osteomyelitis with epidural abscess of the thoracic spine in the setting of *E. rhusiopathiae* septicemia with endocarditis involving the aortic, mitral, and tricuspid valves.

## Case presentation

A 71-year-old homeless male with a history of severe spinal stenosis status-post L4 laminectomy (six weeks prior to presentation), chronic leukocytosis, chronic obstructive pulmonary disorder (COPD), type 2 diabetes mellitus, and hypertension presented with three days of left thumb weakness and thoracic back pain, accompanied by dyspnea and increased sputum production. The pain was described as sharp and constant without radiation, and worse when lying flat on his back. He denied trauma, muscle strain, incontinence, saddle anesthesia, fevers, chills, sick contacts, or recent travel. The back pain and left-hand weakness were first noted on waking three days prior. On presentation, he was afebrile, tachycardic at 124 beats per minute, had a respiratory rate of 17 breaths per minute, a blood pressure of 138/68 mmHg, and was saturating at 95% SpO_2_ on room air. On physical exam, he was resting, in no acute distress, and coughing intermittently. He had a regular rhythm and no murmurs were auscultated. His lungs were clear to auscultation bilaterally, and the abdomen was soft and non-distended. There was bilateral 1+ pitting edema to the lower extremities. No visible skin lesions were noted. His grip strength was 2/5 on the left and fully intact on the right.

Laboratory studies were remarkable for white blood cell count 18.7 × 103/μl (baseline about 14 × 103/μl), hemoglobin 12.7 g/dL, and a negative respiratory viral panel. Plain films of the chest as well as of the thoracic and lumbar spine were unremarkable.

**Figure 1:**
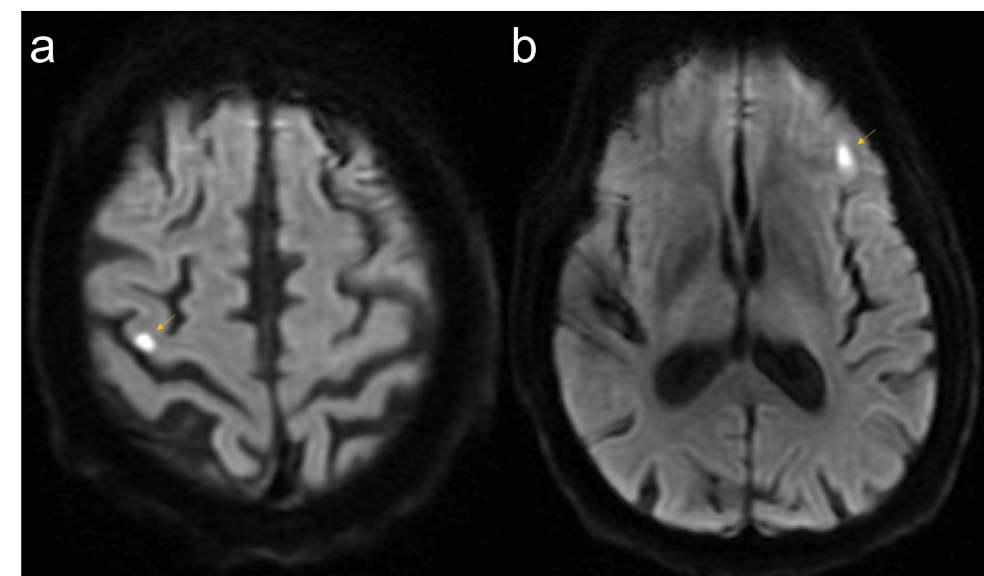
MRI brain without contrast, DWI, axial view. (a) Area of increased signal within the right frontal lobe in the region of the precentral gyrus (arrow) concerning for subacute infarction. (b) Area of increased signal diffusion within the left frontal lobe (arrow) concerning for subacute infarction. MRI: magnetic resonance imaging; DWI: diffusion-weighted imaging.

The patient had a negative bilateral lower extremity venous duplex for deep vein thrombosis (DVT) and a negative computed tomography angiography (CTA) for pulmonary embolism. He was started on a five-day course of prednisone 40 mg daily with nebulized ipratropium/albuterol for suspected COPD exacerbation. Neurology and neurosurgery were consulted for thumb weakness and back pain in the setting of a recent laminectomy. Magnetic resonance imaging (MRI) of the brain without contrast demonstrated a subacute infarct in the right frontal lobe in the region of the precentral gyrus, which correlated clinically with symptom onset four days prior (Figure 1). This also showed an area of increased signal diffusion within the left frontal lobe, possibly representing subacute infarction (Figure 1). Per neurology consultants, these findings were suggestive of bilateral cortical strokes of embolic origin. He was started on aspirin 81 mg daily.

Two days into the admission, the patient was found to have a significant hemoglobin drop from 9.5 g/dL over 24 hours to 6.7 g/dL. This was attributed to a duodenal ulcer, known from upper endoscopy three months prior to admission, which had been positive for the presence of *Helicobacter pylori*. Treatment at that time had been deferred until outpatient follow-up with gastroenterology, given the documentation of positive Shiga-toxin-producing *Escherichia coli* concomitantly. He was transfused with two units of packed red blood cells and was started on quadruple therapy for *H. pylori* infection.

**Figure 2:**
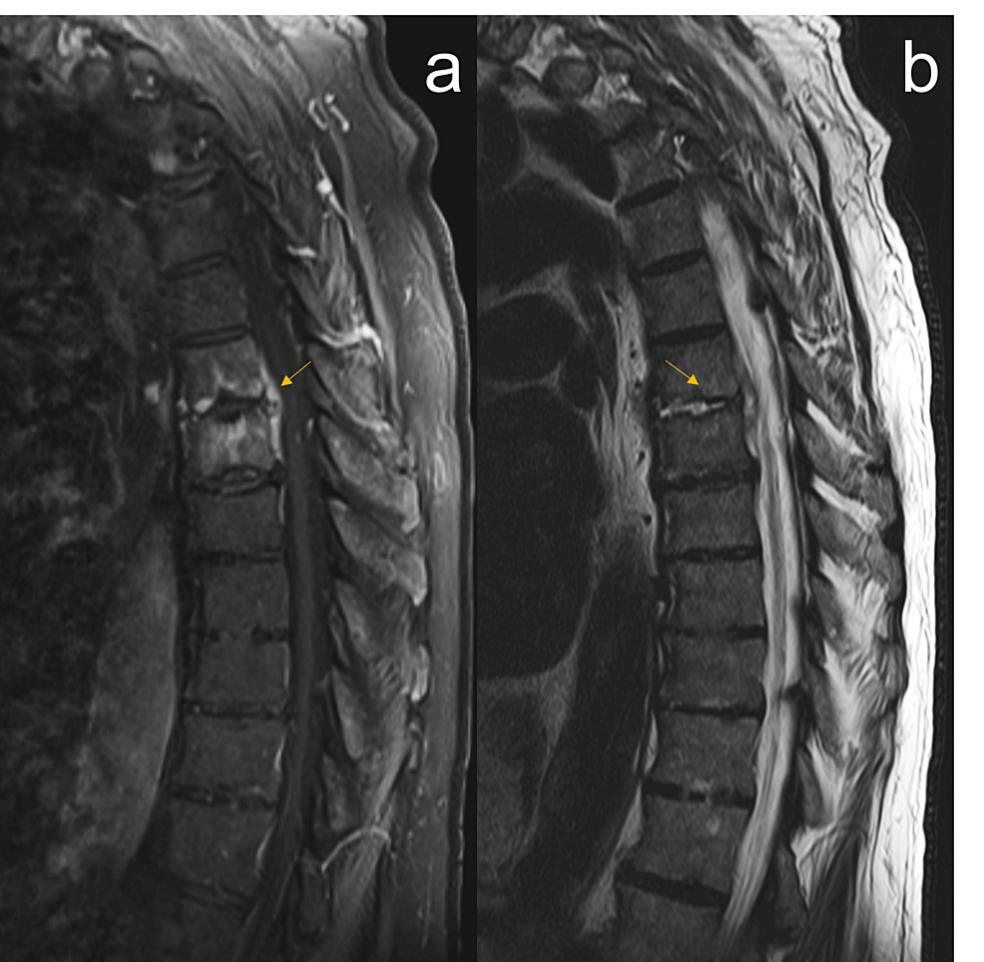
MRI thoracic spine with and without contrast, sagittal view. (a) T1-weighted FS demonstrating increased paraspinal soft tissue enhancement extending into the anterior epidural space with a six-millimeter collection along the left anterior epidural space concerning for a small epidural abscess at T6-T7 (arrow). (b) T2-weighted image demonstrating increased signal at T6-T7 disc space (arrow) associated with bone marrow edema and increased bone marrow enhancement compatible with acute discitis and osteomyelitis. MRI: magnetic resonance imaging; FS: fat saturation.

He then underwent an MRI of the thoracic spine without contrast, which was remarkable for T6-T7 acute discitis with osteomyelitis, as well as a 6 mm fluid collection along the left anterior epidural space, representing a T6-T7 epidural abscess (Figure 2). Vertebral bone biopsy and abscess drainage were performed, and he was started empirically on intravenous (IV) vancomycin every 12 hours (q12h). Blood cultures from admission then grew Gram-positive rods in aerobic bottles, prompting a consult for infectious disease. Initial differential for Gram-positive rods implicated in bone and joint infections included the genera Arcanobacterium, Bacillus, Corynebacterium, Erysipelothrix, and Tropheryma. To complete empiric coverage pending speciation, cefepime 1 g IV q12h was added to the patient’s antibiotic regimen. The original blood culture, as well as subsequent blood cultures, were then speciated into *Erysipelothrix rhusiopathiae*, and he was transitioned from vancomycin and cefepime to ceftriaxone 2 g IV every 24 hours (q24h). Cultures demonstrated sensitivity to penicillin, ampicillin, levofloxacin, ciprofloxacin, erythromycin, and clindamycin.

**Figure 3:**
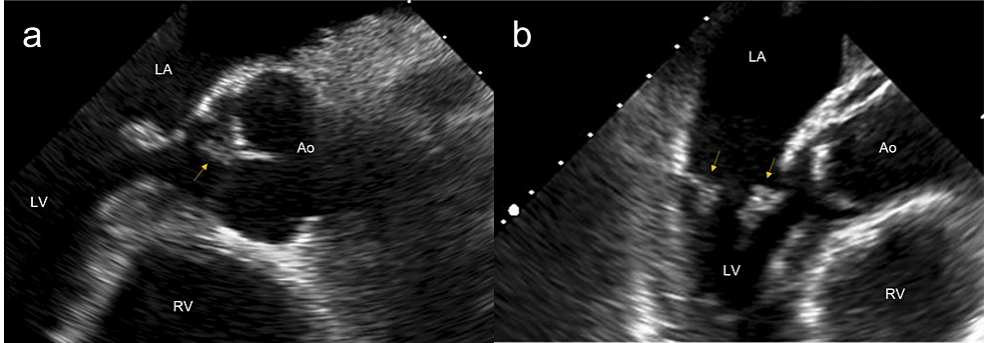
Trans-esophageal echocardiogram. (a) Mid-esophageal long-axis view with a vegetation on the atrial aspect of the aortic valve (arrow). (b) Mid-esophageal 3-chamber view with vegetations on both leaflets of the mitral valve (arrows). LA: left atrium; LV: left ventricle; RV: right ventricle; Ao: aorta.

An initial transthoracic echocardiogram performed at the beginning of the hospitalization and prior to speciation had been unremarkable. However, a repeat study was recommended given the ongoing bacteremia, a known tendency of *E. rhusiopathiae* to cause endocarditis, and MRI findings indicative of recent ischemic stroke with a likely embolic source. This did not show any clear signs of endocarditis. However, the presence of vegetation could not be accurately assessed due to poor image quality. Considering the risk of morbidity and mortality associated with valvular destruction, a follow-up trans-esophageal echocardiogram (TEE) was then pursued. The TEE showed masses consistent with vegetation on both anterior (9.0 mm × 4.8 mm, non-mobile) and posterior (6.3 mm × 4.2 mm, mobile) mitral valve leaflets, as well as on the right coronary cusp of the aortic valve (6.8 mm × 3.3 mm) (Figure 3). Additionally, a small tricuspid valve vegetation versus a focally thickened leaflet could not be excluded. These findings were accompanied by mild mitral regurgitation, trace aortic regurgitation, and mild tricuspid regurgitation. Cardiothoracic surgery stated that no surgical intervention was indicated. He was subsequently placed at a skilled nursing facility for the administration of IV antibiotics for six weeks and continued care. A follow-up TEE six weeks post-discharge was recommended to assess for valvular destruction but was never completed.

## Discussion

In the above case, we present a novel case of *E. rhusiopathiae* septicemic infection presenting with neurologic symptoms from a subacute stroke, likely a complication of embolic phenomena from endocarditis. While most described cases of *E. rhusiopathiae* (Table 1) are linked to common reservoirs such as swine, fish, or crab as part of occupational exposure or due to meat consumption, our patient denied any exposure to fish, raw meats, or other common occupational hazards such as working in a slaughterhouse [2,3]. The patient did, however, endorse that he was housed in poor conditions with rats and raccoons that may have either bitten him in his sleep or had indirect inoculation via flea bite. It is also possible that the patient may have encountered the bacterium via contaminated foods. Other cases have also noted exposure via cat or dog bites and inoculation via a goose rib, though none specifically notes zoonotic infection from rats or raccoons [13-15]. Nevertheless, this patient’s presentation exemplifies that *E. rhusiopathiae* should be included in the differential diagnosis of Gram-positive rod bacteremia in patients with unsanitary housing regardless of known or suspected exposure to common reservoirs of infection.

**Table 1:** Overview of reported cases of Erysipelothrix rhusiopathiae bacteremia with multi-system septic involvement.

Case, year	Patient age (years)	Patient sex	Relevant comorbidities	Initial presentation	Septic systemic involvement	Exposure	Treatment
This report	71	M	Homelessness, diabetes mellitus, recent laminectomy	Left hand weakness, thoracic back pain	Acute osteomyelitis with epidural abscess of the thoracic spine, endocarditis, embolic stroke	Unknown- possible rats, raccoons, Homeless.	Ceftriaxone
Tan et al. [6]	48	M	Fisherman occupation	Blurred vision, dizziness	Endocarditis, embolic stroke	Wild fish handling	Ceftriaxone
Wilson et al. [9]	71	M	Fisherman occupation, coronary artery disease, mitral regurgitation, hypertension	Fatigue, chills	Endocarditis, lumbar spine osteomyelitis	Snow crab handling	Ceftriaxone
Joo et al. [10]	56	F	Steroid use for idiopathic thrombocytopenic purpura, systemic lupus erythematosus	Fever, altered mental status	Endocarditis, meningitis	Unknown	Ceftriaxone
Ko et al. [5]	63	F	Fisherwoman occupation, alcohol abuse, hepatitis B,	Fever, altered mental status	Endocarditis, embolic stroke	Cut finger while handling fish	Penicillin-G, ceftriaxone
Ruiz et al. [11]	76	M	Aortic valve replacement, gout, hypertension.	Left knee pain and swelling	Endocarditis, septic arthritis	Fishing hobby	Penicillin-G
Artz et al. [12]	46	M	Butcher occupation, alcohol abuse	Arthralgias, left arm weakness, left lower extremity paresis, dizziness	Endocarditis, embolic stroke, embolic splenic infarction	Butchering	Amoxicillin/clavulanate, clindamycin
Romney et al. [7]	67	F	Diabetes mellitus	Low-back pain	Lumbar spondylitis, endocarditis	Raw fish handling	Penicillin-G

*E. rhusiopathiae* exhibits likely intrinsic resistance to vancomycin, a highly unusual feature in Gram-positive bacilli. It also appears to have significant resistance to aminoglycosides and trimethoprim/sulfamethoxazole. Sensitivity testing in the literature as well as case reports documenting successful resolution favor prompt treatment with penicillin G, cephalosporins, quinolones, carbapenems, and clindamycin [16,17].

As described previously, *E. rhusiopathiae* is a rare human infection from zoonotic sources with no recorded cases of human-to-human transmission. Its most common manifestation is a localized cutaneous rhomboid cellulitis termed "erysipeloid" due to its gross similarity to erysipelas despite deeper cutaneous involvement. This form of infection is self-limited, lasting three to four weeks without treatment. Less common is the diffuse cutaneous form, where the lesions extend beyond the initial site to other remote places on the body, is accompanied by systemic symptoms such as fever, and lasts longer than the localized form [1,2]. There is limited literature support in favor of antibiotic treatment of the diffuse cutaneous form to shorten the symptomatic duration, but infection is typically self-limited [18].

Septicemic disease is the rarest and most deadly form of infection. Endocarditis is the most common complication of septicemic disease, with a preference for left-sided heart valves. Additionally, mortality associated with *E. rhusiopathiae* endocarditis is as high as 38% and can result in heart failure in up to 80% of cases, making rapid recognition and treatment imperative [3]. While the infection is highly destructive to the valve structure, with up to 36% ultimately requiring valve replacement, no special guidance for surgical management is currently proposed or supported by the literature [3]. Empiric treatment of endocarditis typically includes penicillin, cephalosporin, or carbapenem, but antibiotics should be narrowed if supported by available sensitivities for a total antibiotic course of four to six weeks [7].

Additionally, our patient presented with osteomyelitis complicated by an epidural abscess. While it is reasonable to suppose osteomyelitis was secondary to septic emboli stemming from the heart, it appears bacteremia alone is sufficient for the spread of infection to the bone [8]. Of note, vancomycin monotherapy is occasionally employed in Gram-positive osteomyelitis and should be broadened to include other agents as above when *E. rhusiopathiae* is in the differential. Here, again, parenteral antibiotics should be administered for six to eight weeks [8].

Lastly, our patient’s presentation with three days of thumb weakness was found to be due to a subacute stroke to the right frontal lobe. While his subacute presentation precluded emergent intervention, his stroke and bacteremia informed the diagnostic work-up of his endocarditis. Though it is possible that the patient’s stroke may have been an independent event, the presence of stroke in both hemispheres, as well as co-presentation with osteomyelitis, is highly suggestive that septic embolic phenomena were responsible for both. As such, this case provides a unique example of combined septic, cerebral, osseous, and cardiac infection which had previously been reported separately (Table 1).

## Conclusions

*Erysipelothrix rhusiopathiae* is a gram-positive bacillus typically associated with zoonotic infections contracted from workplace exposure to swine and seafood. Though most commonly manifested as a localized cutaneous form of the disease, termed erysipeloid, it can also be seen to have systemic involvement. It can even go on to involve several organs through hematogenous spread and septic embolism. There have been previously reported cases of *E. rhusiopathiae* bacteremia resulting in endocarditis, osteomyelitis, or septic embolic stroke, among other complications. However, this is the first reported case of all three such sequelae occurring in a single patient. Given its potential for morbidity, it is important to consider this organism in the differential diagnosis of Gram-positive rod bacteremia even in the absence of clear animal exposure, as was the case with our patient.
